# Genotype–Phenotype Discordance in Cardiomyopathies: Pathophysiology, Clinical Expression, and Therapeutic Considerations

**DOI:** 10.1002/hsr2.72398

**Published:** 2026-05-18

**Authors:** Abubakar Nazir, Atif Hussain Sarwar, Rameen Nazar, Sarim Hassan Shahab, Sumera Manan, Hafsa Rafique, Ahsan Talal Khan, Abdalhakim Shubietah, Syed Rafay Hussain Zaidi, Behram Khan

**Affiliations:** ^1^ Department of Internal Medicine The Jewish Hospital—Mercy Health Ohio USA; ^2^ Department of Internal Medicine Oli Health Magazine Organization Kigali Rwanda; ^3^ Department of Internal Medicine Duke University Durham North Carolina USA; ^4^ Department of Internal Medicine Bahria University of Health Sciences Campus Karachi Pakistan; ^5^ Department of Internal Medicine Nishtar Medical University Multan Pakistan; ^6^ Department of Internal Medicine Chandka Medical College Larkana Karachi Pakistan; ^7^ Department of Internal Medicine FMH College of Medicine and Dentistry Lahore Pakistan; ^8^ Department of Internal Medicine Khyber Medical College Peshawar Pakistan; ^9^ Department of Internal Medicine Advocate Illinois Masonic Medical Center Chicago Illinois USA; ^10^ Department of Internal Medicine UC Health Parkview Medical Center Colorado USA; ^11^ Department of Internal Medicine Rochester Regional Health Rochester New York USA

**Keywords:** cardiomyopathy, epigenetics, expressivity, genetic variability, genotype–phenotype correlation, penetrance, precision medicine

## Abstract

**Background:**

Cardiomyopathies encompass a spectrum of myocardial disorders often attributed to underlying genetic mutations. However, genotype–phenotype discordance where the genetic profile does not align with the expected clinical presentation poses significant diagnostic, prognostic, and therapeutic challenges.

**Objective:**

To review the mechanisms underlying genotype–phenotype discordance in cardiomyopathies, explore its clinical implications, and propose future research directions to bridge the gap between molecular findings and patient outcomes.

**Methods:**

A narrative review was conducted using PubMed, Scopus, and Embase for articles published between 2000 and 2025. Search terms included “genotype‐phenotype discordance”, “cardiomyopathy”, “penetrance”, “expressivity”, “modifier genes”, and “epigenetics”. Included studies focused on genetic and clinical variability in hypertrophic, dilated, arrhythmogenic, and restrictive cardiomyopathies. Priority was given to cohort studies, family‐based analyses, and mechanistic investigations addressing epigenetic, polygenic, and environmental contributors to phenotypic heterogeneity.

**Results:**

Multiple factors contribute to genotype–phenotype discordance in cardiomyopathies, including incomplete penetrance, variable expressivity, epigenetic regulation, modifier genes, and environmental influences such as exercise and comorbidities. This discordance complicates risk stratification and personalized therapy. Clinically, individuals with pathogenic variants may remain asymptomatic, while others without identified mutations may exhibit overt disease. Emerging tools such as polygenic risk scores, multi‐omics integration, and longitudinal phenotyping are improving phenotype prediction.

**Conclusion:**

Genotype–phenotype discordance in cardiomyopathies underscores the complexity of translating genetic insights into clinical practice. A multidimensional approach combining genetic, molecular, and environmental data is essential to refine diagnosis, guide management, and inform genetic counseling. Future research should focus on deep phenotyping, systems biology, and longitudinal cohort studies to unravel the dynamic interplay between genes and environment.

## 1 Introduction

Genotype is defined as the individual's variant of a DNA sequence. A phenotype is a person's observable characteristics, which are the manifestation of their genotype and include traits such as height, eye color, and blood type [1]. Sometimes, the genetic variant or genotype is associated with a particular disease, for example, BRCA1 (genotype) is associated with breast cancer (phenotype) [2].

Cardiomyopathies are a group of diseases that affect the muscles of the heart, leading to structural and functional deterioration of the heart [3]. There are several types, including DCM (dilation of the left ventricle) [4], HCM (characterized by left ventricular hypertrophy) [5], ARVC (replacement of right ventricular myocardium with fibrofatty tissue) [6], RCM (increased stiffness of ventricular walls leading to diastolic dysfunction) [7], and LVNC (characterized by layers of myocardium—the compacted and non‐compacted—also known as spongy myocardium) [8]. Genotype–phenotype discordance occurs when the genotype does not match the expected phenotype. It is also referred to as incomplete penetrance. For example, the MHY7 gene for the sarcomere may be mutated, but does not necessarily cause HCM [9]. Approximately 30%–40% (or more) of mutation carriers do not express the phenotype [10].

Genotype–phenotype discordance limits precision medicine, which relies on genetic data to predict disease risk. It can lead to unnecessary interventions and overestimation of disease risk [11]. ICDs are frequently advised based on family history or genotype, particularly in cases of arrhythmogenic cardiomyopathy or HCM. Variants that are not fully penetrant may result in either undertreatment (silent mutation carriers who may still be at risk) or overtreatment (ICD implantation in asymptomatic individuals unlikely to develop the disease) [12]. It also affects family screening, as families may experience anxiety or make poor decisions due to imprecise risk assessments and resulting uncertainty [10].

While several studies have explored various aspects of cardiomyopathy, the literature lacks sufficient information about the interplay of genotype–phenotype discordance and cardiomyopathy. This review aims to consolidate existing knowledge on genotype–phenotype discordance in cardiomyopathy with a focus on mechanisms, diagnostics, research challenges, clinical implications, solutions, and future directions.

## 2 Landscape of Genotype–Phenotype Relationships in Cardiomyopathy

### 2.1 Traditional Monogenic Models

Cardiomyopathies have traditionally been considered monogenic disorders. According to traditional monogenic models, a mutation in a single gene is sufficient to cause disease. These are inherited in a Mendelian fashion. The majority are autosomal dominant, though some forms may be recessive or X‐linked. This model assumes a direct and predictable link between a pathogenic variant and a clinical phenotype. Examples include hypertrophic cardiomyopathy (HCM), which is due to autosomal dominant mutations in sarcomeric genes such as MYH7 and MYBPC3; dilated cardiomyopathy (DCM), which is associated with variants in genes like TTN, LMNA, and TNNT2; and arrhythmogenic cardiomyopathy, which involves desmosomal genes such as PKP2 and DSP [13, 14, 15] (Table 1).

**Table 1:** Genotype–phenotype discordance in cardiomyopathies—Key examples and contributing factors.

Gene/Variant	Typically associated phenotype	Reported discordant phenotypes	Clinical features/Variability	Penetrance/Expressivity	Contributing factors
MYH7	Hypertrophic cardiomyopathy (HCM)	Dilated cardiomyopathy (DCM), left ventricular noncompaction (LVNC)	Phenotypic heterogeneity even within the same family	Penetrance varies (50%–100%)	Modifier genes, environmental triggers
LMNA	Dilated cardiomyopathy with conduction disease	Isolated arrhythmias, sudden cardiac death, skeletal myopathies	Includes AF with slow ventricular response, muscular dystrophies (e.g., limb‐girdle, Emery–Dreifuss), contractures	Incomplete; earlier/more severe in males	Age, sex‐specific expression, epigenetics
PKP2, DSP	Arrhythmogenic cardiomyopathy (ACM)	Biventricular cardiomyopathy, DCM	Desmosomal mutations causing atypical phenotypes	Variable expressivity	Environmental modifiers (e.g., endurance exercise)
TTN (truncating)	Familial DCM	Detected in 1%–3% of healthy individuals	Associated with 25% familial and 18% sporadic DCM; variable onset	Incomplete penetrance, especially in females	Age‐related expression, polygenic background
SCN5A	Brugada syndrome, conduction disease	DCM, overlap syndromes	Multiple arrhythmic/cardiomyopathic phenotypes	Variable	Possibly linked to channelopathies and hormonal factors
SCN10A	Cardiac conduction modulation	Brugada syndrome, conduction delay	Mechanism not fully understood; interaction with SCN5A	Unknown	Expression levels lower than SCN5A in myocardium
Multiple Genes (e.g., TNNI3K, CALM3, ACE2)	Modifier roles in HCM/DCM	Associated with disease severity and onset	SNPs show differential prevalence in affected individuals	Not independently causative	Likely act as phenotypic modifiers
General observation	—	Variable phenotypes despite identical mutations	Clinical expression ranges from asymptomatic to severe disease	Highly variable; influenced by genotype, age, sex	Modifier genes, epigenetics, environmental stressors

The traditional models suggest high penetrance. However, in light of recent clinical data, the monogenic framework is viewed as insufficient to fully explain the complex inheritance patterns of cardiomyopathies. Clinical data have revealed considerable variability in phenotypic expression, disease severity, and age of onset, even among individuals carrying the same pathogenic variant [16].

### 2.2 Discordant Presentations in Known Genes

Ever‐increasing clinical data highlight discordant phenotypic presentations among individuals carrying pathogenic variants in cardiomyopathy genes. This means that mutations in these cardiomyopathy genes can result in unexpected phenotypes, variable expression, different disease severity/progression, or sometimes no disease at all.

For instance, mutations in MYH7, typically associated with HCM, have also been implicated in DCM and left ventricular noncompaction (LVNC), even within the same family [16, 17]. Similarly, LMNA mutations, which usually result in DCM with conduction system disease, may present as isolated arrhythmias or sudden cardiac death in the absence of overt structural abnormalities [18]. Mutations in the LMNA gene can lead to a wide variety of diseases, ranging from premature aging to myopathies and DCM [14] (Table 1).

In cardiomyopathies mediated by LMNA mutations, the presenting feature may be arrhythmias, including atrial fibrillation with a slow ventricular response or ventricular arrhythmias [14]. LMNA mutations may present with or without muscle disease, with muscle disorders ranging from limb‐girdle muscular dystrophy to Emery–Dreifuss muscular dystrophy. Clinical features may include contractures of the elbow and Achilles tendon [14] (Table 1).

Variants in desmosomal genes like PKP2 and DSP, known to cause ACM, have also been reported in patients with biventricular or dilated phenotypes [15]. TTN truncating mutations, a major cause of familial dilated cardiomyopathy, have shown incomplete penetrance and variable expressivity and are also present in 1% of healthy individuals [19]. According to a cohort study of 312 individuals with DCM, truncating mutations in TTN were associated with 25% of familial and 18% of sporadic DCM. However, the study also revealed that 3% of control participants carried these mutations [13].

These different manifestations draw attention to the complex interaction of modifier genes, environmental factors, and epigenetics in clinical expression. The identification of a known pathogenic variant does not imply a predictable phenotype, thus presenting challenges for genetic counseling and risk stratification. Understanding the nature of this variability is critical to realizing the potential of genotype‐led diagnostics and precision medicine in the management of cardiomyopathies.

### 2.3 Incomplete Penetrance and Variable Expressivity

The major contributors to genotype‐phenotype discordance in cardiomyopathies are incomplete penetrance and variable expressivity. Penetrance is defined as the proportion of individuals carrying a particular gene variant who also express the associated trait (phenotype) [13]. Expressivity refers to the degree to which a phenotype is expressed by individuals with a particular genotype [13].

In HCM, due to mutations in MYH7, penetration may exceed 80%–100% in some families, while in others it may be less than 50% [20]. This variability complicates clinical risk prediction and genetic counseling. LMNA and TTN mutations, which result in dilated cardiomyopathy, may show incomplete penetrance, often leading to underdiagnosis in asymptomatic carriers [18].

Sex‐specific effects have been reported. Males with LMNA or TTN variants often exhibit earlier onset and more severe cardiac dysfunction than females, suggesting hormonal or physiological factors [21]. There are also age‐related aspects of penetration. Typically, a carrier's disease expression increases with age, though many carriers do not express an overt phenotype until mid‐life or later [22]. Incorporating incomplete penetrance and variable expressivity is essential to effectively and accurately optimize clinical management and develop precision‐based approaches for inherited cardiomyopathies.

## 3 Mechanistic Basis of Discordance

### 3.1 Modifier Genes

Hypertrophic and dilated cardiomyopathies (HCM and DCM) are characterized by marked genetic and clinical heterogeneity, suggesting the involvement of modifier genes and environmental factors in their pathophysiology. A study evaluating the association of single nucleotide polymorphisms (SNPs) in three candidate genes ACE2 (*rs6632677*), TNNI3K (*rs49812611*), and CALM3 (*rs13477425*), with clinical phenotypes in North Indian patients demonstrated significant genotype–phenotype correlations. The ACE2 variant was notably more prevalent among cases compared to controls. Additionally, the homozygous genotypes of TNNI3K (3784 C > T) and CALM3 (−34T > A) were significantly enriched in both HCM and DCM patients [23].

Voltage‐gated sodium channel gene SCN10A variants are implicated in cardiac conduction abnormalities and Brugada syndrome. However, the precise mechanisms by which SCN10A subtypes contribute to dysfunctional cardiac conduction remain unclear. Studies have shown that SCN5A expression is significantly higher in both human and murine hearts compared to SCN10A, suggesting differential functional roles [24].

Mutations in sarcomeric protein‐encoding genes represent the most common genetic basis of cardiomyopathies. The genetic architecture of HCM has been found to be more complex than previously appreciated. These mutations can remain clinically silent or manifest with variable symptoms and outcomes, complicating genotype–phenotype correlations [25].

Variants in SCN5A have been associated with cardiomyopathic phenotypes. Specifically, the non‐functional SCN5A A735E mutation has been linked to a spectrum of cardiomyopathic presentations, implying the influence of unidentified modifiers in phenotypic variability [26].

Drug‐induced cardiomyopathies further highlight the interplay of genetic and environmental factors. Agents such as anthracyclines, tyrosine kinase inhibitors (TKIs), immune checkpoint inhibitors (ICIs), fluoropyrimidines, and VEGF inhibitors exert cardiotoxic effects through diverse molecular pathways. These effects are modulated by individual susceptibility, sex, ethnicity, metabolic response, and drug dosage. Genetic variants associated with drug‐induced cardiotoxicity are categorized into common (minor allele frequency > 5%) and rare (< 0.1%) variants, both of which may predispose to cardiotoxic outcomes [27].

Mutations in SCN5A have also been implicated in DCM, particularly those affecting voltage‐sensitive domains. Screening of DCM cohorts has identified five pathogenic SCN5A missense mutations: D1275N, F1520L, V1279I, E446K, and R222Q [28].

Over the past decade, more than 100 mutations have been identified in 11 core sarcomeric genes associated with HCM. Additional mutations have been found in genes encoding subunits of AMP‐activated protein kinase, mitochondrial DNA, and those involved in triplet repeat syndromes. Notably, significant phenotypic variation exists even among patients with identical pathogenic mutations, as demonstrated in genotype–phenotype correlation studies [29].

### 3.2 Epigenetics

Cardiomyopathies encompass a diverse group of myocardial disorders characterized by structural, functional, and pathological alterations of the cardiac muscle. While genetic mutations play a central role in the initiation and progression of cardiomyopathies (CMPs), emerging evidence highlights the significant influence of epigenetic and environmental factors in modulating disease expression and clinical outcomes.

Epigenetic mechanisms, including DNA methylation, histone modifications, chromatin remodeling, and noncoding RNAs, have been implicated in the pathogenesis and progression of CMPs. These modifications can alter gene expression without changes in the underlying DNA sequence and are increasingly recognized as both potential diagnostic biomarkers and therapeutic targets. For instance, the use of histone deacetylase (HDAC) inhibitors is under investigation as a strategy to reverse maladaptive gene expression profiles in heart disease. Importantly, these epigenetic changes are modifiable and are often influenced by environmental exposures such as diet, toxins, and stress [30].

The interplay between genetic and epigenetic regulation has been elucidated through advanced experimental models, including intergenerational inheritance studies and monozygotic twin analyses, which demonstrate significant variability in phenotype despite identical genotypes. This suggests that non‐genetic modifiers substantially contribute to disease penetrance and expressivity [30].

Notably, the burden of cardiomyopathy is rising in Asian populations, even as it appears to be stabilizing or declining in the West. Recent studies in Asian cohorts have uncovered unique combinations of rare and common genetic variants. Epigenomic profiling, particularly DNA methylation studies in heart failure, have provided compelling evidence that these modifications underlie regional and individual variations in clinical presentation and severity [31].

In the context of DCM, mortality rates are rising in some regions, often in association with increased ventricular dilation and systolic dysfunction. While the genetic basis of DCM is well established, recent data reveal a substantial contribution of epigenetic dysregulation. For example, novel DNA methylation patterns have been associated with DCM, including alterations in genes such as *Lymphocyte Antigen 75 (LY75)* and *Adenosine Receptor A2A (ADORA2A)*, validated in zebrafish models. These findings underscore the pathogenic significance of methylation‐mediated gene silencing or activation [32].

Additionally, histone modification abnormalities have been observed in left ventricular tissue from patients with non‐ischemic DCM. Specifically, there is a notable reduction in histone marks such as H3K4 trimethylation (H3K4me3) and H3K9 dimethylation (H3K9me2), suggesting that chromatin remodeling contributes to transcriptional misregulation in the failing heart [32].

Epigenetic mechanisms are also responsive to physiological and pathological stimuli, including alcohol consumption, psychological stress, nutritional status, physical activity, and smoking. These external factors can induce rapid and sometimes reversible epigenetic modifications, highlighting the dynamic and interactive nature of genetic and environmental contributions to cardiomyopathy pathophysiology [33].

### 3.3 Environmental and Lifestyle Triggers

ACM is characterized by progressive fibrofatty replacement of the myocardium, which may affect either the right, left, or both ventricles. Unlike the general population, in whom physical activity confers cardiovascular and psychological benefits, individuals with ACM do not derive such advantages from exercise. On the contrary, physical activity, particularly high‐intensity endurance sports has been shown to accelerate disease progression in ACM, likely through both structural remodeling and enhanced arrhythmic risk [34].

Rest and reduced physical exertion have been associated with a slower disease course, and current evidence suggests that the intensity of exercise, rather than its duration, plays a more critical role in determining outcomes. Vigorous physical activity induces myocardial adaptations that mimic an athlete's heart physiology, which may worsen structural and electrophysiological abnormalities in ACM patients. Clinical guidelines recommend limiting physical activity to < 6 metabolic equivalents (METS) and < 2.5 h per week for both symptomatic patients and mutation carriers to reduce arrhythmic risk [34, 35].

Ventricular arrhythmias are a hallmark of ACM and are frequently precipitated by exercise, which supports the heightened risk of sudden cardiac death (SCD) in athletes. Patients with ACM or at‐risk genotypes are strongly advised to avoid competitive or high‐intensity sports [35] (Table 2).

**Table 2:** Mechanistic basis of genotype–phenotype discordance in cardiomyopathies.

Mechanism	Genetic/Clinical elements	Key examples	Clinical implications
Modifier genes	Influence expression/severity of primary mutations	SNPs in ACE2 (rs6632677), TNNI3K (rs49812611), CALM3 (rs13477425) found associated with HCM/DCM in North Indian patients; SCN10A and SCN5A interaction affects conduction and arrhythmias	Modifiers alter penetrance/expressivity; can mask or exaggerate phenotypes
Epigenetics	DNA methylation, histone modifications, chromatin remodeling, noncoding RNAs	DNA methylation changes (e.g., in A2A, LY75) and histone marks (e.g., H3K4me3, H3K9me2) in DCM; emerging role in Asian populations	Epigenetic marks may serve as biomarkers or therapeutic targets; modifiable by environment
Environmental and lifestyle triggers	Exercise, drugs, infection, pregnancy, autoimmune disease	ACM exacerbated by vigorous exercise; myocarditis from viral infections or ICIs progressing to DCM; case of SCD in pregnant athlete with RVOT‐VT and MYBPC3 variant	Lifestyle/activity can unmask genetic risk; gene‐exposure interactions influence disease onset
Somatic mosaicism and de novo mutations	Mutations arising post‐zygotically or spontaneously in germline	De novo MYH7 mutation (p.L908P) causing early‐onset HCM; ACTC1/TTN co‐mutation in early DCM; TAZ mutations causing Barth syndrome	Early‐onset or atypical presentations; require trio testing or early surveillance (e.g., ICD)
Gene–gene and gene–environment interactions	Combined effects of multiple genes or gene + environment	TTN + toxin exposure in DCM; FLNC, LMNA, DSP variants with environmental modifiers; monozygotic twins with MYH7 HCM show differing severity	Explains discordant phenotypes in families; requires polygenic and environmental profiling
Inflammation and secondary disease triggers	Viral, autoimmune, or drug‐related myocardial injury	Myocarditis‐induced DCM; ICIs and sarcoidosis‐mediated cardiomyopathy; cardiac MRI findings in DSP mutation carriers	Inflammatory or autoimmune triggers can mimic or worsen inherited forms; imaging + genetics critical
Stem cell models/functional insights	hiPSC‐CMs used for functional validation of variant effects	hiPSC‐derived cardiomyocytes from ACM patients show stress‐response and regeneration capacity	Tool for mechanistic research and individualized therapy validation

A notable case highlighting the fatal potential of ACM involved a professional female athlete in the 36th week of gestation, previously diagnosed with right ventricular outflow tract ventricular tachycardia (RVOT‐VT). Despite a successful ablation 4 years prior and an uneventful pregnancy, she experienced sudden cardiac arrest during labor. A healthy infant was delivered via emergency cesarean section, but the mother did not survive. Autopsy revealed extensive fibrofatty infiltration of the myocardium. Genetic testing revealed a variant of uncertain significance in the *MYBPC3* gene, though no definitive association with arrhythmogenic right ventricular cardiomyopathy (ARVC) was established in this case [36].

Myocarditis is another significant environmental trigger that can lead to the development of DCM through structural and functional myocardial damage. The acute inflammatory phase is marked by myocyte necrosis and interstitial fibrosis, which can lead to ventricular dilation and systolic dysfunction. Even after resolution, residual scar tissue and adverse remodeling may persist. Infectious agents such as cytomegalovirus are well‐established etiologies of chronic myocarditis and resultant DCM. Importantly, studies have shown that effective infection control may prevent or limit the progression of this complication [37].

In addition to infections, autoimmune‐mediated inflammation can also contribute to DCM pathogenesis. Chronic immune responses targeting cardiac myocytes may lead to ongoing injury and impaired contractility. Immune checkpoint inhibitors (ICIs), commonly used in oncology, have been associated with immune‐mediated myocarditis, characterized by lymphocytic infiltration and myocyte necrosis, resulting in progressive myocardial weakening. Furthermore, adrenal insufficiency, systemic inflammatory diseases such as sarcoidosis and rheumatic fever, and myocardial involvement in autoimmune disorders can all lead to inflammatory cardiomyopathies that clinically mimic or transition into DCM [37] (Table 2).

### 3.4 Somatic Mosaicism and De Novo Mutations in Cardiomyopathy

Dystrophin, a critical structural protein, plays an essential role in maintaining the integrity of the sarcolemmal membrane and myofilament alignment during cardiac muscle contraction. Mutations in the *DMD* gene, which encodes dystrophin, are causative of Duchenne Muscular Dystrophy (DMD) and are frequently associated with early‐onset cardiomyopathy and heart failure. Variants in *DMD* and *TNNI3K* genes have been implicated in severe pediatric phenotypes of cardiomyopathy, suggesting a key role in early disease pathogenesis [38].

Genetic screening of families affected by cardiomyopathies has revealed pathogenic variants in core sarcomeric genes. In one study, two deleterious heterozygous mutations *ACTC1* (c.664 G > A; p.Ala222Thr) and *TTN* (c.33250 G > A; p.Glu11084Lys) were identified through next‐generation and in silico analyses. A de novo mutation in *ACTC1*, co‐expressed with a *TTN* variant, was proposed as the underlying cause of early‐onset, severe DCM, and premature mortality [39].

HCM is the second most common form of cardiomyopathy in infants, with SCD being a frequent fatal outcome. In a documented case, a de novo heterozygous *MYH7* mutation (c.2723 T > C; p.L908P) led to HCM with disrupted protein structure and function. Prophylactic implantation of an implantable cardioverter‐defibrillator (ICD) in this pediatric patient was successful, with no reported complications at discharge, highlighting the importance of early genetic diagnosis and intervention to prevent SCD in children with pathogenic mutations [40].

Mutations in the *TAFAZZIN* (TAZ) gene, which encodes an acyltransferase critical for cardiolipin biosynthesis, are responsible for Barth syndrome (BTHS), a disorder characterized primarily by cardiomyopathy. The cardiac phenotype of BTHS has been well‐replicated in *TAZ* knockout mouse models, supporting the translational relevance of these mutations to human disease [41] (Table 2).

### 3.5 Gene–Gene and Gene–Environment Interactions

Knowing further detail about different DCM‐associated genes, gene‐specific DCM outcomes, and information about various environmental interactions have helped in better understanding of inherited DCM. Associations of genes with DCM, gene‐specific DCM outcomes, and variant environment interactions has greatly improved our understanding of inherited DCM. The current genotype–phenotype profiling has identified a new, DCM‐associated FLNC variant, and another factor, LMNA, that helps in prognosis of DSP inflammatory cardiomyopathy. Genetic factors, such as the role of TTN variants, and the environmental factors on toxin‐mediated DCM [42].

It has been seen that monozygotic twins had mutations of MYH7 along with hypertrophic cardiomyopathy, using methods of exome and Sanger genetic sequencing. This study shows the relation with the pathophysiological mechanism of HCM and the impact of epigenetics and environment [43]. Patients with cardiomyopathies present with complex clinical presentations; it has become easier with the help of genetic testing to understand the genetic association in this behavior. Cardiac inflammation has been seen on cardiac MRI, and further genetic testing revealed a pathogenic mutation in the desmoplakin gene, which is present in ACM [44].

ACM has a strong genetic basis. Patients with this present with arrhythmic episodes. Changes in different genes like PKP2, JUP, DSC2, DSG2, and DSP are responsible for desmosome‐related ACM. This causes sudden cardiac death in athletes, and with longer standing, it causes fibrofatty infiltration. Improvements have been seen with human‐induced stem cells; these cardiac myocytes can bear the changes because they have the potential to expand and come back to the original state after stress disappears. DNA of cells produced by hiPSC is the same as that from those from which it was derived [41].

## 4 Diagnostic and Research Challenges

### 4.1 Limitations of Genetic Testing

Variants of unknown significance (VUS) in genetic testing present significant diagnostic challenges in cardiomyopathies associated with genotype–phenotype discordance. In a study, 70% of VUS were present on genes without strong evidence of disease association [45]. It leads to misinterpretation of genetic results and hinders accurate risk assessment [45] and causes confusion regarding patient prognosis and management of patients with discordant clinical presentations [46]. In a recent study involving 170 patients, 19.8% of positive patients had C4 or C5 variants, whereas 73.1% of patients were classified as C3‐VUS, emphasizing the prevalence of uncertain findings in genetic testing and management issues [47]

One of the limitations in genetic testing is incomplete penetrance. It complicates the interpretation of genetic testing, resulting in cardiomyopathies with genotype–phenotype discordance. Cardiomyopathies show complex inheritance beyond dominance pattern, and variability in penetrance makes it difficult to forecast the course of disease [48]. In hypertrophic cardiomyopathy, penetrance varies significantly, ranging from 11% to 57% [49]. Not all variant carriers will manifest disease, and those who do, it significantly varies with age and can't be reliably predicted [49, 50]. Small cohorts usually lack the diversity and statistical power needed to accurately classify genetic variants and often misclassify benign variants as pathogenic due to their low frequency and thus overrepresent them as pathogenic even though they are benign in a large population [51]. Similarly, testing in ethnic minorities may lead to underrepresentation and exacerbate limitations in genetic testing and accurate diagnosis [52]. Low ancestral diversity in genetic studies over the period of time may contribute to misclassification and diagnostic uncertainty in variant assessments [53].

### 4.2 Role of Imaging and Biomarkers in Resolving Discordance

Use of cardiovascular magnetic resonance (CMR) has enabled characterization of myocardium without needing invasive biopsy techniques [54]. CMR not only provides information on cardiac morphology, function, and tissue characterization but also quantification of myocardial wall thickness [55]. It is a diagnostic and prognostic tool in hypertrophic cardiomyopathy, identifying hypertrophy in even those areas that are blind to echocardiography [56]. Contrast‐enhanced CMR with Late Gadolinium Enhancement (LGE) assesses myocardial fibrosis or scar burden [57, 58] and provides additional information for assessing SCD event risks. The extent of LGE is a strong predictor of SCD events [59]. LGE has been used as a reference standard for the differential diagnosis of ischemic [60] and non‐ischemic cardiomyopathies [61]. Diffuse fibrosis, which is undetected by LGE imaging, is detected and quantified by parametric mapping methods, that is, T1 mapping [62]. Contrast‐ enhanced T1 mapping is also used for calculating the Extracellular Volume (EVC) fraction. Tissue characterization via Native T1 mapping is an important diagnostic, therapeutic, and prognostic factor in various cardiac diseases [62].

Assessment of Global longitudinal strain (GLS) has been shown as a predictive biomarker in cardiomyopathies, particularly in DCM [63, 64] and HCM [65]. GLS being more sensitive than left ventricular ejection fraction (LVEF) can reveal subclinical myocardial dysfunction [66]. Investigation of GLS in subclinical carriers, particularly in genotype–phenotype discordance, reveals significant insights into early myocardial dysfunction. Asymptomatic individuals carrying genetic variants associated with DCM often exhibit reduced GLS, indicating subclinical systolic dysfunction despite having a normal ejection fraction (LVEF) [67]. Presence of reduced GLS in genotype‐positive, phenotype‐negative individuals indicates a significantly higher risk of developing overt DCM, suggesting close monitoring and early intervention for these patients [68]. Biomarkers like troponins, Brain Natriuretic Peptide (BNP), and high‐sensitivity C‐reactive protein provide insight regarding myocardial injury, stress and inflammation, and play a crucial role in early detection of cardiomyopathies. Troponin levels can be chronically raised and detected in subclinical myocytes damage [69]. High levels of troponin and BNP have been associated with cardiomyopathies, particularly HCM [69, 70, 71], suggesting more chances of adverse cardiovascular events in these patients [72]. Thus, biomarker measurements can be used as a pretest in selecting patients for imaging techniques for the early management of CM [73].

### 4.3 Ethical and Counseling Considerations

Genotype–phenotype discordance in cardiomyopathies presents significant ethical and counseling challenges, particularly in genetic counseling and patient management. Complexities of genetic interactions and their variability in phenotype expression impact the severity and prognosis of disease [74]. A significant number of individuals with pathogenic variants don't develop overt disease, complicating clinical management [75]. It necessitates careful implication of results to patients and their families, considering potential benefits and risks of disclosing uncertain and non‐penetrant findings, as disclosure of uncertain findings can cause anxiety and surveillance fatigue in patients [76]. An ethical dilemma of disclosing results to biological relatives without consent and cascade testing may also arise while considering the implications of genetic findings for family members who may be at higher risk of developing the disease [76].

## 5 Clinical Implications of Discordance

The discordance between genotype and phenotype in cardiomyopathies poses significant challenges in clinical decision‐making. Emerging evidence underscores the need for personalized approaches to risk prediction, surveillance, and therapy.

### 5.1 Sudden Cardiac Death in Mutation‐Positive, Phenotype‐Negative Individuals

Genotype‐positive individuals without overt structural cardiomyopathy face a significant risk of SCD, challenging traditional risk stratification paradigms. This clinical dilemma is well‐illustrated in a conceptual framework that outlines the management complexities of genotype‐positive but phenotype‐negative individuals [77]. Pathogenic variants in LMNA and PLN exemplify this discordance. In a large five‐generation family with a novel LMNA nonsense mutation (c.544 C > T), 17 SCDs occurred at a mean age of 49.3 ± 10.0 years, often preceding left ventricular dysfunction. Affected individuals exhibited progressive conduction abnormalities (AV block, atrial fibrillation) and ventricular arrhythmias, with SCD occurring in unprotected patients despite preserved ejection fraction (EF) [78, 79].

Similarly, PLN p.Arg14del mutation carriers demonstrate a high arrhythmic burden, with a prediction model identifying male sex, late gadolinium enhancement on cardiac MRI, and inferolateral T‐wave inversions as key risk factors for malignant ventricular arrhythmias [80]. Long‐term data from a multicenter cohort confirmed the variable penetrance and clinical heterogeneity in PLN R14del carriers, reinforcing the need for tailored risk stratification [81].

Current ICD guidelines lack consensus for asymptomatic carriers. While ESC 2022 recommends prophylactic ICD implantation in LMNA carriers with PR interval > 240 ms or nonsustained ventricular tachycardia (NSVT), AHA/ACC 2023 guidelines restrict this to patients with reduced EF [78, 82]. For PLN p.Arg14del carriers, risk stratification integrates clinical, imaging, and genetic data, with a validated prediction model achieving 85% accuracy in identifying high‐risk individuals [80]. Desmosomal gene variants (e.g., DSP, PKP2) in DCM patients are associated with a 4.4% annual risk of SCD or ventricular arrhythmias, independent of EF [82]. These findings underscore that arrhythmic risk in genotype‐positive individuals is not solely dependent on structural remodeling, necessitating revised surveillance protocols.

### 5.2 Risk Stratification Tools

Emerging machine learning (ML) algorithms enhance risk prediction in genotype‐positive cardiomyopathy patients. A 2024 study demonstrated that ML models integrating ECG data, genetic variants, and cardiac MRI parameters improved arrhythmia prediction accuracy to 89% compared to traditional clinical scores [83]. For example, a deep learning‐based ECG risk score (DL‐ECG) predicted long‐term cardiovascular death with a C‐index of 0.83, outperforming conventional metrics like LVEF [84].

ECG‐genotype correlations are increasingly validated: LMNA mutation carriers exhibit low‐voltage QRS complexes (specificity: 92%), while PLN variants correlate with inferolateral T‐wave inversions [83]. Advanced tools like AI‐driven ECG‐to‐CMR translation models can non‐invasively detect subclinical structural abnormalities (e.g., fibrosis) in mutation carriers with normal echocardiograms [85].

### 5.3 Tailored Surveillance and Management

#### 5.3.1 Age‐ and Sex‐Specific Surveillance Protocols

PLN R14del mutation carriers exhibit age‐dependent phenotypic expression, with symptom onset typically in mid‐adulthood, variable penetrance, and possible earlier arrhythmia onset. Regular cardiac screening (ECGs, Holter monitoring, echocardiography) is recommended starting at age 20–30 years, with frequency adjusted by clinical risk. Some data suggest male carriers may experience arrhythmic events earlier, though sex differences are not strongly emphasized. For LMNA cardiomyopathy, early surveillance, possibly beginning in adolescence, may be warranted due to the risk of early‐onset conduction disease, as observed in a three‐generation family with progressive atrioventricular block [79]. Sex‐specific considerations include pre‐pregnancy cardiac evaluation, guided by arrhythmia and conduction status, to manage hemodynamic risks during pregnancy.

#### 5.3.2 Use of Wearable Tech in Early Detection

Although not directly evaluated in the cited studies, wearable technologies (e.g., smartwatches with ECG capabilities) align with proactive monitoring trends. These devices may enhance arrhythmia detection in high‐risk groups, such as identifying transient atrial fibrillation in LMNA carriers or ventricular arrhythmias in PLN mutation carriers, prompting timely clinical review.

#### 5.3.3 Guidelines for Sports Participation and Pregnancy

Guidelines suggest caution or restriction from competitive sports in mutation carriers with arrhythmic risk, especially those with known conduction disease or left ventricular dysfunction. For PLN carriers, moderate exercise may be permitted if ventricular function is preserved; LMNA carriers with conduction abnormalities should avoid strenuous activity. During pregnancy, multidisciplinary care is essential. Pre‐pregnancy device evaluation and programming optimization may be considered to reduce hemodynamic risk in LMNA carriers, while preconception arrhythmia and left ventricular function assessments are critical for PLN carriers.

## 6 Emerging Solutions and Future Directions

### 6.1 Polygenic Risk Scores and AI‐Based Prediction Models

DCM is mainly related with genetic basis, it has been shown that familial DCM is a monogenic disorder, its variant shows autosomal dominant inheritance patterns. Further studies and genetic testing help to understand both pathogenic and non‐pathogenic variants, making researchers go in detail and learn the genetic architecture.

### 6.2 iPSC‐Based Disease Modeling and CRISPR Screens

A total control cohort of 1127 individuals was used for comparison due to the restriction that data from the above study is only present on request. These committees approved the study (St. Vincent's Hospital Human Research Ethics and Melbourne Human Research Ethics). All individuals were of European ancestry. DCM‐associated variants, both pathogenic and non‐pathogenic were 48%. The Mann–Whitney U test was used for differences in percentile distributions. It provides a common overview of genetic variations in suspected monogenic disease [86].

DCM is more prevalent in cardiomyopathy 1 in 250–400 individuals. New discoveries in the treatment and diagnosis of disease have shown better disease prognosis and patient survival. Association of DCM with different genetic variants (with a minor allele frequency (MAF) of more than 1%) is supported by two studies: genome‐wide association studies (GWAS) and polygenic risk score (PGSs). DCM diagnosis can be made with a dilated and poor contractile ventricles. It depends on whether DCM is present in a single person or is also present in other family members. We need clinical features only for isolated cases in the family, but if this is accompanying other family members, genetic screening would be preferred. Conventionally, it's been reported that Mendelian inheritance is responsible. Others are pathogenic variants of disease‐associated genes. The ClinGen consortium has found 19 genes responsible for DCM, such as TTN, LMNA, and BAG3. Genome‐wide association studies want to look at genetic changes and results. GWAS can be widely used for many types of genetic variations, copy‐number, and rarer sequence genes. It mainly focuses on single nucleotide polymorphisms (SNPs) and small insertions and deletions [87].

In the entire world, the largest number of patients with peripartum cardiomyopathy is in Nigeria. A clinical trial randomized in pregnant and postpartum women has been conducted by applying AI‐guided or informal techniques, so that it can assess the impact on LVSD in the period from 22 weeks of gestation till postpartum period. Different tools were used, like digital stethoscope recordings and a 12‐lead electrocardiogram with asynchronous AI predictions for LVSD. Results proved that no participation‐related unwanted effects were present. AI‐guided screening that utilized the stethoscope has positive effects on the diagnosis of pregnancy‐related cardiomyopathy [88]. New innovations and discoveries make AI capable of drawing models and maps that would precisely categorize cardiomyopathy patients from non‐cardiomyopathy patients. These advancements are helping in identifying risks and diagnosing disease, so that on‐time identification of cardiomyopathic patients can occur even without symptom appearance. These models have various capabilities, can use clinical data sets, for example, electrocardiogram recordings, cardiac imaging, and other multi‐modal genetic and omics data sets (Table 3).

**Table 3:** Emerging solutions and future directions.

Category	Description	Tools/Technologies
Study population and methodology	1127 individuals (European ancestry) studied; Mann–Whitney U test used to assess genetic variant distribution; study approved by ethics committees (St. Vincent's Hospital and Melbourne Human Research Ethics).	Ethics‐approved cohorts; statistical testing (Mann–Whitney U)
Genetic associations in DCM	DCM occurs in 1 in 250–400 individuals; GWAS and PGSs identified 19 associated genes (e.g., TTN, LMNA, BAG3); focus on SNPs and copy‐number variations; importance of clinical screening in familial vs. isolated cases.	GWAS, PGS, SNPs, CNVs, ClinGen gene panels
AI and cardiomyopathy screening	AI‐based tools (e.g., digital stethoscopes, ECGs) used in Nigerian cohort; trial in pregnant and postpartum women showed safe and effective diagnosis of peripartum cardiomyopathy.	AI diagnostics, digital stethoscopes, ECG, asynchronous models
iPSC and CRISPR‐based modeling	iPSC and CRISPR used to model cardiovascular diseases; genome editing facilitates mutation discovery; accelerated research in gene regulation and epigenetics.	CRISPR‐Cas9, iPSC, next‐generation sequencing
Pluripotency and iPSC generation	iPSCs derived from adult somatic cells using Yamanaka factors (OCT3/4, SOX2, KLF4, c‐MYC); modern delivery methods (episomal plasmids, synthetic mRNAs) reduce genomic integration risks.	Yamanaka factors, episomal plasmids, synthetic mRNA
Long‐read sequencing (nanopore)	Genes like MYBPC3, MYH7, TNNT2 analyzed via long‐read Nanopore sequencing and Long Range PCR; shown to outperform Sanger in coverage and mutation detection.	Oxford Nanopore, Long Range PCR, Nanopolish, Clair3
Structural variant detection	Detected deletions, duplications, inversions in congenital heart disease (CHD); long‐read GS showed 16% diagnostic rate; Illumina paired analysis used.	Illumina sequencing, long‐read genome sequencing, paired analysis
Transcriptome analysis	RNA‐seq and transcriptome sequencing revealed causative splice variants in HCM and muscle disorders; 35% diagnostic yield in genetically unsolved cases; full‐length transcript analysis improved mutation identification.	RNA‐seq, transcriptome sequencing, splice variant analysis

#### 6.2.1 IPSC‐ Based Disease Modeling and CRISPR Screens

CVS are most common problems causing major mortality and morbidity. Models such as the mouse model helped to study and understand genetic, epigenetic, and signaling pathways. It is further assisted by CRISPR genome editing, like next‐generation sequencing technologies, which have sped up the process of mutation identification. Similarly, it is true in the case of gene editing in mice modeling and iPSC. Earlier, almost 30 years ago, enzymes were found in fungi, useful for developing site‐specific double‐stranded breaks. Later, it was copied, and different nucleases were being produced. CRISPR Case is one of them, which has greatly made the system easy, such as genome editing. With further advancement, CRISPR genome has been used for gene regulation, epigenetic modification, and chromatin imaging [88, 89].

The development of case‐specific cardiomyocytes for disease modeling or regenerative therapy commonly utilizes induced pluripotent stem cells (iPSCs) derived from adult somatic cells. Reprogramming of these somatic cells is achieved by introducing Yamanaka transcription factors OCT3/4, SOX2, KLF4, and c‐MYC, which restore pluripotency and enable differentiation into any cell type derived from the three germ layers, including cardiomyocytes [90, 91].

Initially, viral vectors were employed to integrate these transcription factors into the host genome. However, integration posed safety risks due to potential insertional mutagenesis and retention of viral elements. To overcome these limitations, non‐integrating approaches have been developed, such as the use of episomal plasmids, synthetic mRNA, and non‐integrating RNA viruses (e.g., Sendai virus), which avoid genomic incorporation and reduce the risk of genetic instability. These technological advancements have significantly improved the safety, efficiency, and clinical applicability of iPSC‐based approaches, particularly in cardiovascular precision medicine (See Table 3).

### 6.3 Long‐Read Sequencing and Structural Variant Detection

#### 6.3.1 Advancements in Long‐Read Sequencing and Transcriptomic Analysis in Cardiomyopathy Diagnostics

Recent developments in genomic technologies have significantly enhanced our ability to detect pathogenic variants associated with hereditary cardiomyopathies. Using Oxford Nanopore Technology (ONT) in combination with Long‐Range PCR and advanced bioinformatic pipelines, researchers have explored key HCM‐related genes such as MYBPC3, MYH7, TPM1, TNNT2, and TNNI3. Two complementary strategies, Nanopore long‐read sequencing for base‐level resolution and long‐range PCR for gene‐specific enrichment, have been employed to interrogate these loci. Compared to traditional Sanger sequencing, second‐ and third‐generation sequencing platforms offer higher throughput, broader coverage, and faster turnaround for detecting complex variants.

When clinical suspicion is limited to a few genes, targeted sequencing using exome hybridization strategies proves efficient. Software tools such as Nanopolish and Clair3 have further streamlined variant calling and interpretation in long‐read data sets. These advances support a growing shift toward third‐generation sequencing for comprehensive diagnosis of inherited cardiac conditions.

Additionally, Illumina short‐read genome sequencing has demonstrated a 16% diagnostic yield in a large cohort of patients with congenital heart disease (CHD), the most common form of birth defect. This approach detected various genomic alterations—including single‐nucleotide variants, deletions, duplications, and insertions—though it remained limited by false‐positive rates in inversion detection.

**Figure 1:**
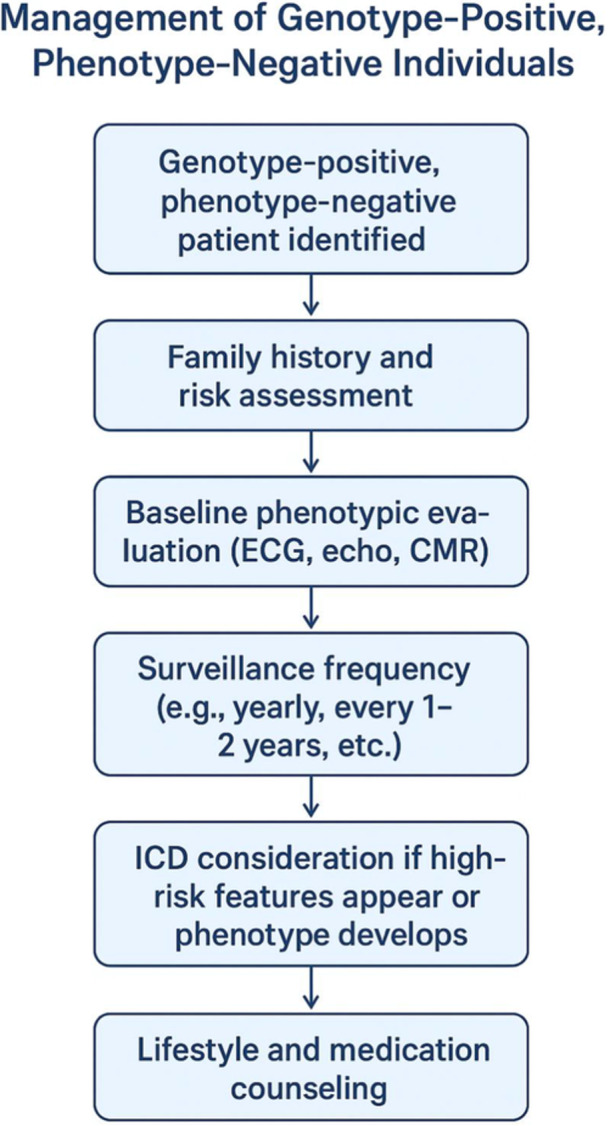
Proposed management algorithm for genotype‐positive, phenotype‐negative individuals.

In parallel, transcriptomic sequencing (RNA‐seq) is increasingly employed to resolve genetically undiagnosed cases. In a large HCM cohort, disease‐associated variants were identified in only 32% of patients through conventional sequencing, with 15% remaining genetically unexplained. RNA‐seq, particularly using long‐read transcriptomics, enables the identification of cryptic splice‐altering variants and provides full‐length transcript coverage. For instance, a putative splice‐site mutation in *MYBPC3* was detected in a female HCM patient using this approach, underscoring its diagnostic utility in previously unresolved cases (Figure 1).

## 7 Conclusion

Genotype–phenotype discordance in cardiomyopathies underscores the complex interplay between genetic, epigenetic, and environmental factors in disease expression. While advances in genomic technologies have enabled the identification of numerous pathogenic variants, translating these findings into predictable clinical phenotypes remains a significant challenge. Incomplete penetrance, variable expressivity, polygenic influences, and external modifiers all contribute to the observed heterogeneity in clinical outcomes. This discordance has critical implications for diagnosis, risk stratification, and therapeutic decision‐making, necessitating a more integrated approach that incorporates genomics, longitudinal phenotyping, and systems biology.

## Author Contributions

Abubakar Nazir was involved in conceptualization, project administration, writing – review, and designing. All authors contributed to writing the first draft, revising, reviewing, and editing. All authors approved the final manuscript and submission.

## Funding

The authors have nothing to report.

## Ethics Statement

The authors have nothing to report.

## Conflicts of Interest

The authors declare no conflicts of interest.

## 1 Peer Review

Not commissioned, externally peer reviewed.

## Transparency Statement

The lead author, Abubakar Nazir, affirms that this manuscript is an honest, accurate, and transparent account of the study being reported; that no important aspects of the study have been omitted; and that any discrepancies from the study as planned (and, if relevant, registered) have been explained.

## Data Availability

The authors have nothing to report.
